# Diagnosis of Mycobacterium marinum Infection Based on Seroconversion of QuantiFERON-TB Gold Test

**DOI:** 10.7759/cureus.9208

**Published:** 2020-07-15

**Authors:** Nagakrishnal Nachimuthu, Santosh Yatam Ganesh

**Affiliations:** 1 Internal Medicine, Catholic Health Initiatives (CHI) St. Luke's Health Memorial, Livingston, USA

**Keywords:** quantiferon-tb, atypical mycobacteria, immunocompromised, mycobacterium marinum

## Abstract

*Mycobacterium marinum *is a slow-growing photochromatic acid fast bacilli (AFB). Following exposure of injured skin to fish tanks and other aquatic bodies, it usually causes indolent skin and soft tissue infections. Incubation period differs but it is generally long; hence, diagnosis is often missed leading to delay in treatment. Obtaining proper history along with histopathology and cultures leads to diagnosis. There is evidence of cross-reactivity of* M. marinum* with QuantiFERON-TB gold test. In patients without risk factors for tuberculosis, recent seroconversion may provide a clue to diagnosis and eliminate differentials. We present a case of *M. marinum* skin and soft tissue infection diagnosed based on seroconversion of QuantiFERON-TB gold test in an immunocompromised patient. This was confirmed by AFB culture after six weeks.

## Introduction

*Mycobacterium marinum* is confirmed with bacterial growth in acid-fast bacilli (AFB) culture. Cultures often take a long time, thus delaying the diagnosis. *Mycobacterium Marinum* infection is often confused with other differentials including *Sporotrichosis*, *Leishmaniasis*, and *Nocardiosis*, infections due to other non-tuberculous mycobacteria (NTM), cellulitis secondary to *Staphylococcal* and *Streptococcal* bacteria, and rheumatoid nodules especially in patients with multiple risk factors and exposures. We report a case of *M. marinum* infection in a rheumatoid arthritis patient with recent QuantiFERON-TB gold seroconversion. We would like to familiarize our readers that due to close genetic makeup with *Mycobacterium tuberculosis* (MTB), *M. marinum* can lead to positive QuantiFERON-TB gold test, and this may be used as a quick diagnostic tool especially when patient has minimal risk factors for tuberculosis [1].

## Case presentation

**Figure 1:**
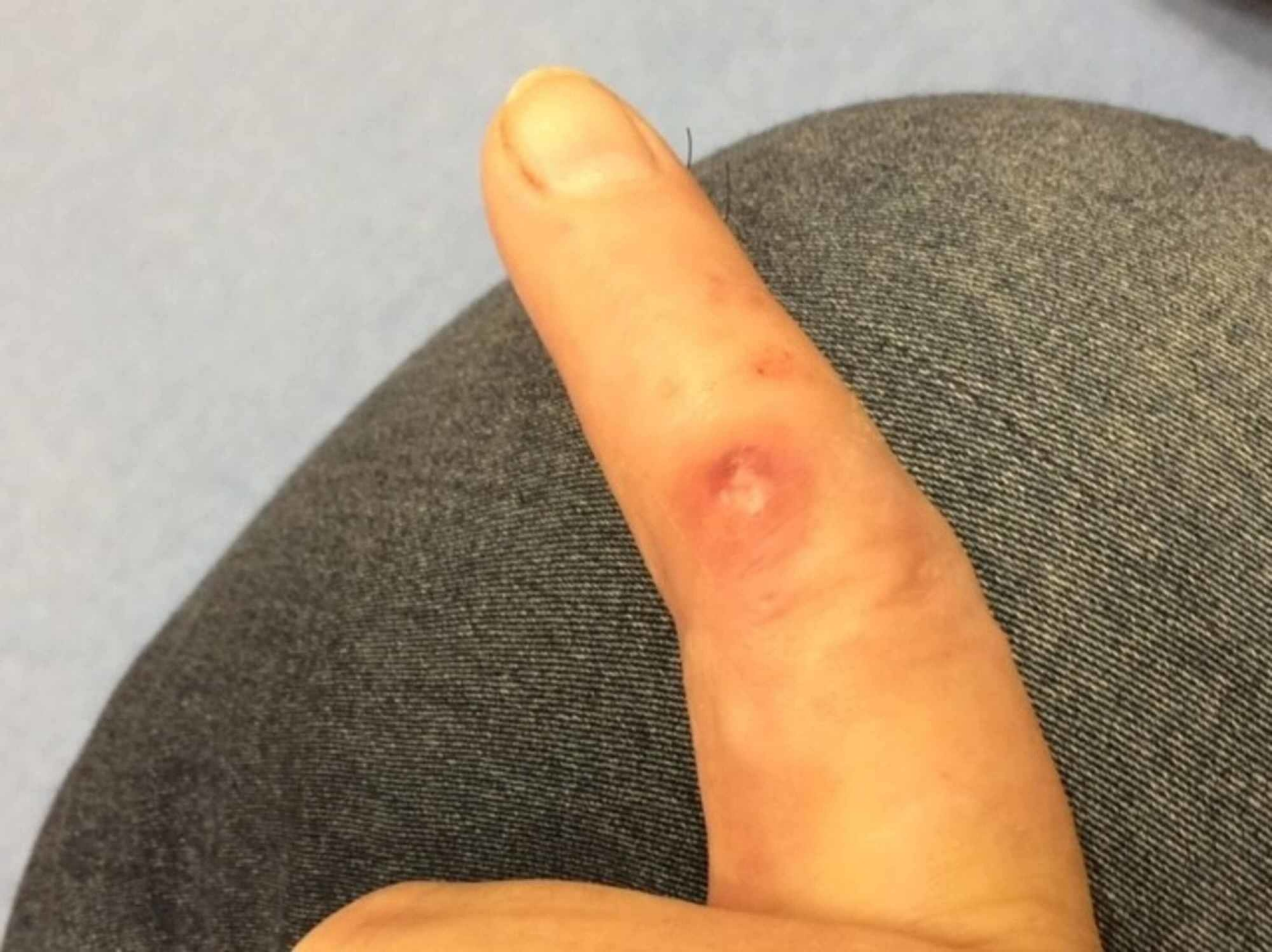
Nodule on the index finger

**Figure 2:**
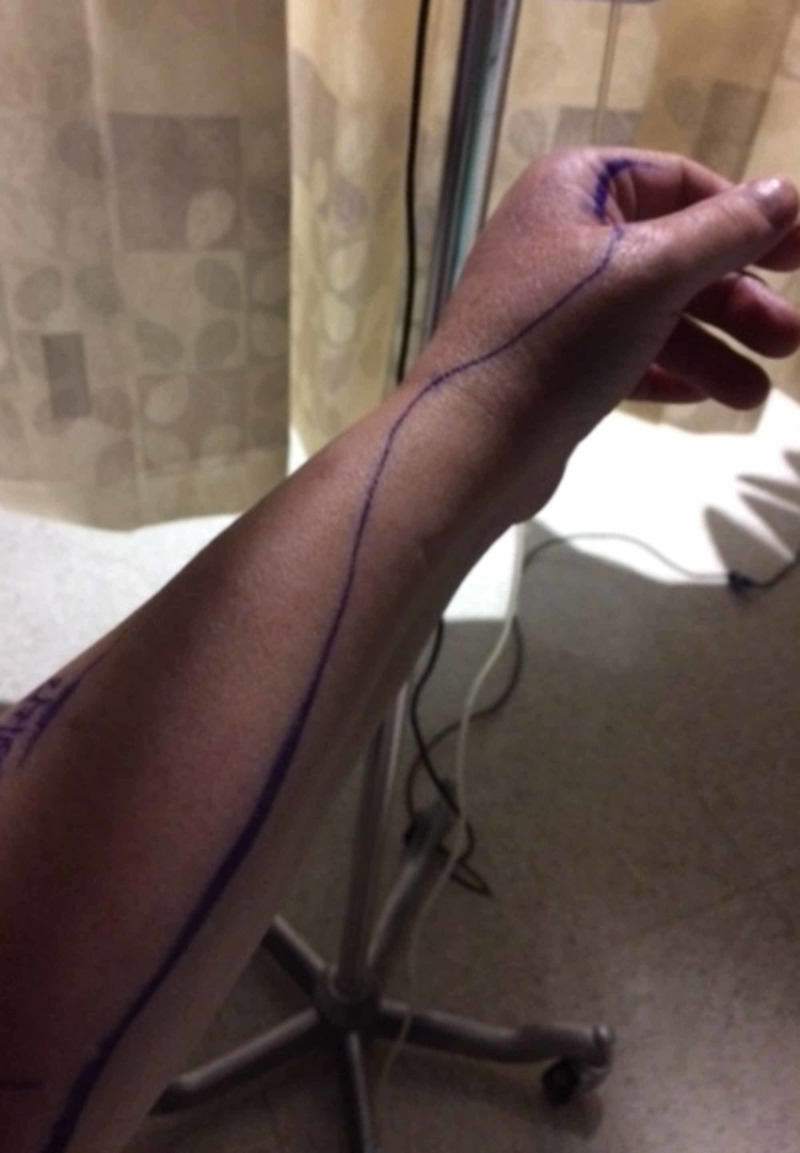
Edema and erythema extending to the forearm while the patient was admitted in the hospital

A 53-year-old female with a history of diabetes and rheumatoid arthritis on golimumab injections was referred from the orthopedic surgeon’s office due to tender nodules with surrounding redness on her left index finger and forearm. Her symptoms initially started about two months back. She reported she was reusing lancets for checking blood sugars and noticed swelling of the left index finger and forearm. Over a period of time, redness along the left index finger and forearm became worse. Her primary care physician prescribed two antibiotics (ceftriaxone IM and dicloxacillin PO). Few days after starting antibiotics, the patient noticed small painful nodules on the index finger and streaking up the forearm (Figure 1). She was referred to the orthopedics department where an ultrasound was performed, which did not reveal any abscess; therefore, she was sent to the Infectious Disease Clinic for further management. Due to failure to respond to oral antibiotics, she was admitted to the hospital (Figure 2), where she was started on vancomycin, ceftriaxone, and clindamycin. The redness and streaking appeared to have improved with antibiotic therapy, and she was discharged on clindamycin and cefdinir for 10 days.

**Figure 3:**
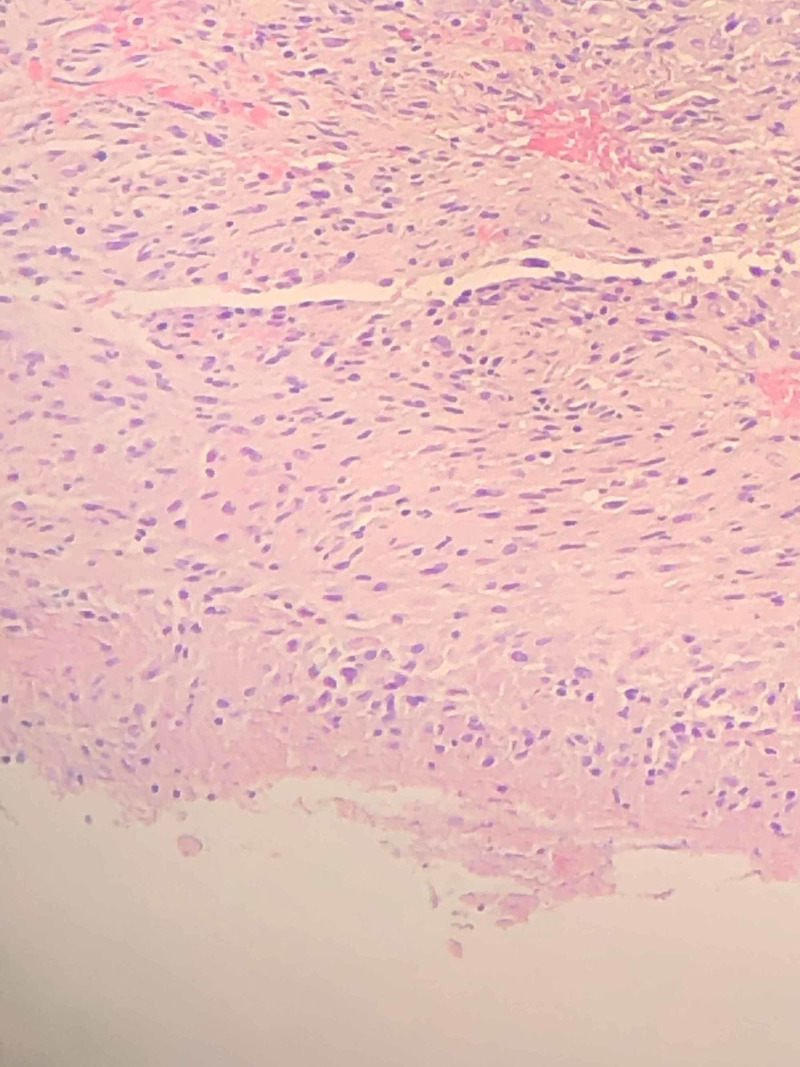
Histological examination revealed part of a granuloma with necrosis and peripheral palisading of fibrohistiocytic cells.

The patient followed up a month later. Her nodules on the left index finger continued to persist. She also informed that her yearly QuantiFERON-TB gold test performed with the rheumatologist had seroconverted to positive. QuantiFERON-TB gold plus results showed a TB1-NIL value of 3.96 IU/mL and a TB2-NIL value of 4.16 IU/mL. The patient always had negative QuantiFERON-TB gold in the past and had no risk factors for tuberculosis. Her chest X-ray was negative. The QuantiFERON-TB gold seroconversion along with persistent nodules gave a clue to diagnosis. Further detailed history revealed that she has a rose garden and admitted to often having injuries with thorns. She also admitted cleaning her fish tank regularly with bare hands. On examination, there was a puncture wound on the left index finger with small nodules, with swelling and erythema extending to the wrist. Labs revealed normal white blood cell count. X-ray of hand revealed no foreign body or acute bony abnormality. The patient underwent biopsy of one of the nodules, which revealed necrotizing granulomatous inflammation (Figure 3). Gram stain, aerobic and anaerobic cultures, AFB, and fungal stain were negative. The clinical history along with recent seroconversion of QuantiFERON-TB gold and evidence of histopathology findings were indicative of *M. marinum* infection. AFB cultures at the end of six weeks confirmed the diagnosis of *M. marinum* infection. The patient was started on minocycline and clarithromycin for nine months. Her nodules resolved completely and did not recur during her one-year follow-up. Once antibiotic treatment was started, she continued receiving monthly golimumab injections.

## Discussion

*Mycobacterium marinum* is an atypical mycobacterium initially reported from fish at an aquarium in Philadelphia [2]. *Mycobacterium marinum* was later noted to be a human pathogen in 1951 in the United States in swimmers using public pools [3]. Initial cluster of cases in humans were reported in swimming pools so originally named as “swimming pool granuloma”; however, with improved disinfection methods, cases were no longer reported in swimming pools but still reported in other natural water bodies. Risk factors include handling of fish either related to occupation or as a hobby [4].

*Mycobacterium marinum* is an AFB that grows well at a temperature of 30 degree Celsius. They are photochromogenic, i.e., colonies are generally white when kept in dark but turn yellow when exposed to light [5]. 

*Mycobacterium marinum* infection occurs following trauma or skin injury and usually after a long incubation period, present as either ulcers or nodules, or can cause deeper and invasive infections such as tenosynovitis and osteomyelitis. Majority of the lesions were found on the upper extremities [6-8].

The diagnosis of *M. marinum* is difficult and often delayed due to insidious clinical presentation. Our case report emphasizes that in patients with low risk of *MTB* infection, a seroconversion of QuantiFERON-TB gold test should raise suspicion for infections with NTM especially *M. marinum*, *M. kansasii*, and *M. szulgai* in appropriate clinical scenario [1]. This case also highlights the importance of obtaining good history and analyzing the laboratory work up in the context of risk factors and exposures.

In a study by Lewis et al., it was found that all patients with *M. marinum* infection were reacting positive to the tuberculin skin test, with reaction more than 10 mm in diameter [9]. Due to various shortcomings with the tuberculin skin test, newer tests were developed that were based on interferon (IFN) gamma production to early secretory antigen target 6 (ESAT-6) and culture filtrate protein 10 (CFP-10) [10]. ESAT-6 and CFP-10 are RD1 (region of difference) that induce IFN-gamma production infected with *MTB* and pathogenic *Mycobacterium bovis* [11]. *Mycobacterium marinum* is closely related to *MTB* in terms of genomic analysis, which could be the reason behind the cross-reactivity to the tuberculin skin test [12].

It is known that there is cross-reactivity to QuantiFERON-TB gold test between *M. marinum*, *M. kansasii*, and *MTB* as they share similar gene code for antigen ESAT-6 and CFP-10, which elicit IFN-gamma from T cells [13]. IFN-gamma quantity is measured in QuantiFERON-TB test. The study conducted by Kobashi et al. in 2006 to re-evaluate the use of QuantiFERON-TB 2G test to differentiate between Active TB and NTM revealed promising results with high positive rate for non-*Mycobacterium avium* complex (non-MAC) NTM comprising mainly of *M. Kansasii *and *M. marinum*, but the absolute number of patients with those diseases were small. In the same study, the results showed low positive rates for MAC [13]. In another study conducted in 2009 for evaluating QuantiFERON-TB Gold test in patients with NTM, it was noted that sensitivity was at 52% and specificity at 93% among non-MAC NTM. As expected, the positive rate was only 1% for MAC. The results indicated that if QuantiFERON-TB test is positive in a patient with low risk of TB, the next possible cause is either *M. marinum* or *M. kansasii* than MAC. It was also noted in the study that if the cutoff value is lowered to 0.20 IU/mL from 0.35 IU/mL used in the test, sensitivity of the test further increases to 82% while the specificity remains high at 91%. Further studies will be needed to evaluate the significance of decreasing cutoff values in aiding the diagnosis of NTM [1]. This indicates that QuantiFERON-TB gold can be used as an ancillary to NTM diagnosis while awaiting AFB cultures and to eliminate differentials such as *Leishmaniasis*, *Sporotrichosis*, *Coccidioidomycosis*, and other fungal infections. This is particularly useful especially when the patient has little to no risk factors for tuberculosis while awaiting AFB cultures.

There are no treatment guidelines for *M. marinum* likely due to lack of clinical trial and it being a rare disease. Treatment has been reported in various cases with different regimens. More commonly used agents are doxycycline or minocycline in combination with co-trimoxazole, rifampin, etc. [14]. Some skin and soft tissue infections may improve spontaneously without treatment [15]. In deeper infections, a longer duration of treatment of up to two years has been reported. For deeper infections, surgery may also be required. Timely diagnosis is necessary for initiating appropriate treatment, especially in immunocompromised patients, as it may lead to disseminated infection particularly with use of immunosuppressive medication [16].

## Conclusions

*Mycobacterium marinum* infection of the skin is rare and can be easily missed. This case describes how recent seroconversion of QuantiFERON-TB gold test led to the diagnosis, thereby ruling out other differentials. Further studies are required to validate the use of QuantiFERON-TB test in diagnosing atypical mycobacterial infections other than MAC and tuberculosis.
